# Autoimmunity (or Not) in Atopic Dermatitis

**DOI:** 10.3389/fimmu.2019.02128

**Published:** 2019-09-10

**Authors:** Lennart M. Roesner, Thomas Werfel

**Affiliations:** Division of Immunodermatology and Allergy Research, Department of Dermatology and Allergy, Hannover Medical School, Hanover, Germany

**Keywords:** autoimmunity, autoallergy, atopic dermatitis (AD), skin, allergy, T cell, IgE, autoreactivity

## Abstract

Atopic dermatitis (AD), one of the most frequent inflammatory skin diseases worldwide, is believed to result from a disturbed skin barrier as well as aberrant immune reactions against *per se* harmless allergens. Starting mostly during childhood with a chronic, remitting relapsing course, the disease can persist into adulthood in about one fifth of patients. Immune reactions to self-proteins have been observed in AD patients already in the beginning of the Twentieth century, when human cellular extracts were shown to provoke skin lesions. However, the term “autoimmunity” has never been claimed, since AD is first and foremost an atopic disease. In contrast, this IgE-hallmarked autoreactivity was termed “autoallergy” and is ongoing discussed regarding its impact on the disease. Since severely affected patients tend to develop IgE-hypersensitivity reactions to numerous environmental allergens, the impact of immune responses to self-proteins is difficult to determine. On the other hand: any autoreactivity, irrespective of the magnitude, implicates the potential of driving the chronification of the disease while shaping the immune response. This review article revisits the observations made on autoallergy from an actual point of view and tries to approach the question whether these still point to a contribution to the disease.

## Autoreactive IgE—an AD-Specific Phenomenon?

First of all, autoreactivity accompanying atopy is a historical observation. The first studies date back to the 1920s, where human skin extract, injected into the skin led to visible skin inflammation (1, 2). These observations have been made long before IgE was discovered (3). In these days, disease criteria and names were not as clearly defined as today, making it somewhat speculative from today's perspective to draw precise conclusions. From what we know today, we can only speculate that in these experiments antigens of the skin dander extracts were captured by recipient's IgE, which was bound to Fc-receptors on the cell surface of mast cells or basophils. This would have led to IgE crosslinking, the release of pro-inflammatory mediators, and finally type I hypersensitivity reactions of the skin.

Serum total IgE levels are often drastically increased in atopic patients and are applied as diagnostic tools and therapeutical targets (4, 5). However, the specificity of the majority often remains unclear. By now, also several modern approaches of controlled experiments focused on the question of autoreactive IgE. Summarizing 14 studies involving 2,644 patients in total, Tang et al. finally conclude that AD is indeed associated with IgE-autoreactivity (6). In this meta-analysis the authors summarize the frequency of affected AD patients to be between 23 and 91% without finding a correlation to age, sex, or disease duration. Interestingly, two of 14 studies detected a significant correlation between IgE-autoreactivity and AD disease severity (7, 8); and in further three studies this became apparent by trend (9–11). As mentioned above, severely affected patients do indeed show strongly elevated total IgE levels, with a bouquet of allergen-sensitizations including e.g., aero-, food-, and microbial allergens. Therefore, it might seem not surprising to find IgE also against self. Nevertheless, autoreactive IgE may play a role in the pathogenicity of the disease, since compared to pollen or food allergens, self-antigens are by nature perennial and inescapable.

The 14 studies mentioned above contained different control entities including other skin autoimmune diseases as psoriasis (8, 10–12) or systemic lupus erythematosus (13), of which none had detectable autoreactive IgE toward the respective autoallergens tested. Nevertheless, autoreactive IgE can be found also in other inflammatory skin diseases [for a review see (14)]: For example, patients suffering from bullous pemphigoid are IgE-(as well as IgG- and IgA-)sensitized against the hemidesmosomal proteins BP180 (BP antigen 2) and BP230 (BP antigen 1). These and other antigens of autoimmune blistering diseases lead to destruction of skin integrity, and the mechanisms are meanwhile quite well understood (15). In systemic lupus erythematosus, patients often display autoreactive IgE to double-stranded DNA or P2 proteins. Recently, an extensive study revealed that autoreactive IgE can be found in the majority of patients with chronic spontaneous urticaria: Reporting over 30 autoreactivities, most prominently the cytokine IL-24 appears as a target for specific IgE in these patients (16). Maurer et al. summarized different studies and come to the point that autoreactive IgE is not exclusively found in AD (14), however, the large number of >140 autoallergens described in this disease up to now appears indeed to be unique (see Table 1).

**Table 1:** IgE-autoantigens (“autoallergens”) described in atopic dermatitis.

**IgE against**	**Prevalence**	**References**
Manganese superoxide dismutase (MnSOD, SOD2)	42%	(8, 17)
Ribosomal protein P2 (RPLP2)	8%	(18)
Profilin 1 (PFN1)	n.a.	(19)
Thioredoxin (TXN, hTrx)	n.a.	(20)
SART-1/Hom s1	n.a.	(19)
α-NAC/Hom s2	30%	[(21); own observations]
BCL7B/Hom s3	n.a.	(21)
MICU1/Hom s4	16.7%	(12, 21)
Cytokeratin 6A/Hom s5	n.a.	(21)
Cyclophilins A, B, C (PPIA, PPIB, PPIC)	n.a.	(22)
Dense fine spreckles (DFS70/LEDGF)	10–30%	(23, 24)
Actin-α	15.5%	(11)
Tubulin-α	21.7%	(11)
eIF6	25.4%	(11)
HLA-DR-α	8.7%	(11)
RP-1	21%	(11)
>124 further antigens	n.a.	(11)

## The Origin of Autoreactive IgE—a Remnant of the Childhood, Crossreactivity, or *de novo* Sensitization?

The frequent occurrence of IgE sensitization to autoallergens in patients with AD was considered as a result of tissue damage and thereby release of auto-antigens that are commonly invisible to T cells (25). Since AD starts in most cases during infancy, several studies investigated autoreactive IgE in children: In a study from 2005, Mothes et al. investigated retrospectively a cohort of 174 adult AD patients regarding the presence of auto-IgE and found 23% to be positive (10). These displayed generally stronger disease symptoms, including clinical signs and scores, increased pruritus, more often a positive history of food allergy, higher levels of total as well as aero-allergen-, food-allergen-, and microbial allergen-specific serum IgE. These patients also reported more frequently to suffer from recurrent bacterial and viral infections of the skin such as impetigo contagiosa or eczema herpeticatum. But most interestingly, an early onset of AD and manifestation of clinically symptomatic AD between the 2nd and 6th years of life was associated with auto-IgE (10). In that work, also sera from 102 children aged 1–12 suffering from AD were analyzed and the authors detected auto-IgE in a substantial subgroup. Children aged 2–13 were affected more often than 1-year-olds. Longitudinal sampling suggested a development of auto-IgE in younger years. However, this study lacks a control cohort of healthy children (10).

In adult patients, auto-IgE in healthy children aged 10–15 was measured by Kistler et al. (26). Samples were generated within the birth cohorts GINIplus and LISAplus and therefore are population-representative. The authors agree with the finding by Mothes et al. that auto-IgE is quite frequently detectable in children of that age, however, the occurrence of auto-IgE was unexpectedly decreased in children suffering from AD and allergic asthma compared to healthies. Therefore, the occurrence of auto-IgE in children appears to be a general phenomenon with so far unknown meaning, but is not a predictor regarding AD. The authors speculate that a general type-2 immune prevalence in early life may be an opposing mechanism to more harmful type-1 (auto)inflammation (26).

Autoreactive IgE antibodies have been identified by detecting interactions between self-antigens and IgE in the serum of patients. In order to define single *Aspergillus* allergens, Crameri et al. established an *Aspergillus fumigatus* phage display library and applied sera of patients with known respective sensitization (17, 18). The discovery of two autoallergens occurred subsequently by investigating sequence homology of the newly identified allergens manganese superoxide dismutase (MnSOD, later termed Asp f6) and ribosomal protein P2 (termed Asp f8) to human proteins. Both of the human homologs, MnSOD and P2 shared strong sequence similarities and subsequent IgE-immunoblotting confirmed a cross-reactivity of the IgE between human and *Aspergillus* proteins. While P2-specific IgE was found in around 8% of 75 AD patients investigated (18), MnSOD sensitization was observed in more than 40% of 69 AD patients tested (8). By comparing results from cDNA libraries that displayed putative allergens from the fungi *Aspergillus* and *Malassezia*, respectively, Limacher et al. came across the thioredoxins that were later termed Asp f28, Asp f29, and Mala s13 (20). Sequence homology led to the identification of the highly homologous human variant, hTrx, and those from further organisms, defining a pan-allergen family. Competetive ELISAs confirmed IgE-crossreactivity, especially between the microorganisms, but also between *Malassezia* and the autoallergen hTrx. *Malassezia* has been known for decades as a trigger factor in AD, colonizing the skin as a facultative pathogen (27). Therefore, a sensitization to Malassezia was suggested to be underlying the cross-reactivity to hTrx, although these hypotheses are difficult to prove.

In direct approaches to identify autoallergens, cDNA phage libraries were generated from human proteins. Therefore, again a crude extract from the human epithelial cell line A431 was applied (21, 28). Binding to full length recombinant and native proteins was validated after recombinant protein expression and (competitive) IgE-blotting experiments. In total, five autoallergens were identified in these fundamental studies that were termed according to the IUIS nomenclature “Homo sapiens allergen 1 to 5” (Hom s1-s5). MICU1/Hom s4-specific IgE was found in a subsequent study to cross-react to homologous proteins of different species, all bearing calcium-binding abilities, namely Phl p7 (timothy grass) and Cyp c1 (common carp) (12). Finally, 10 years ago, a comprehensive phage display approach mapped in total 140 bona fide autoallergens, while confirming 16 that had already been described (11).

In order to approach the question of clinical relevance, recombinantly produced versions of several autoallergens were successfully tested toward IgE-reactivity in patient's skin by means of prick testing [Hom s2, s3, s4, s5 (21) and the ribosomal protein P2 (18)]. While this approach underlines the clinical relevance of auto-IgE, the sensitization to autoallergens and the mechanism of cross-reactivity was further addressed *in vivo*. Upon sensitization with α-NAC/Hom s2, mice developed skin symptoms as well as specific crossreactive IgE and IgG antibody responses. Intradermal administration of the autoallergen led to skin symptoms in sensitized mice as well as in non-sensitized mice after passive transfer of serum of sensitized ones (29).

Regarding the specificity of allergen extract-based IgE-binding assays, one has to consider that also in healthy individuals certain irrelevant cross-reactive carbohydrate antigens bind IgE without mounting a pro-inflammatory response (25). Further, some studies assume that IgE antibodies show a strong potential of crossreactivity, binding to a wide range of epitopes (30). This implicates that identified autoreactive IgE may not be a result of an interaction between B and autoreactive T helper cells. Therefore, the T cell response to putative autoallergens has to be investigated, too, in order to draw a comprehensive picture.

## Autoreactive T Cells as a Result of Crossreactivity or *de novo* Sensitization?

T helper cells of the Th2 subtype are capable of initiating the class switch in B cells to induce the production of antigen-specific IgE. In AD, the T cell response is together with skin barrier disturbance regarded as the central disease mechanism. T cells home to the skin in AD patients (31), and those isolated from the inflamed, lesional AD skin have been shown to react to environmental allergens (32). It has been observed that during an ongoing acute or chronic AD inflammation, skin-infiltrating T cells are mostly T helper cells. Nevertheless, CD8^+^ T cells are also present and furthermore, these have been described to be crucial in initiating the skin inflammation (33, 34). Regarding T cell polarizations, first of all Th2, but also Th1, Th17, and Th22 T cells have been described to contribute to the pathogenesis of AD (35). These appear to be a result of (a) the allergen, (b) the inflammatory milieu, and/or (c) the disease progression. To explain this heterogeneity, it has been proposed that the Th1-predominance in chronic AD lesions might be a result of T cell responses to non-classical allergens like autoallergens. However, analyzes of autoallergen-specific T cell responses confirm this theory only partially.

Again, the primary observations regarding autoreactive T cells in AD have been made astonishingly long ago. Hashem et al. reported 1,963 proliferation of lymphocytes after stimulation with autologous skin extracts, detected by analyzing cell shape and division (36). In these tests, two patients with severe eczema reacted stronger compared to two healthy controls and one asthmatic. The first observations by Crameri et al. on T cell reactions toward the self-antigen MnSOD were made on T cells from AD donors sensitized to fungal and human MnSOD. However, that time no control donors were compared (17). A second trial showed in a proof-of-concept approach that non-sensitized AD patients do not show T cell proliferative responses to human-, *Aspergillus-* or *Malassezia*-MnSOD (8), while in a third approach also healthy controls next to sensitized and non-sensitized AD patients were enrolled in T cell proliferation studies (37). The human ribosomal P2 protein led to T cell responses in six out of six senzitized AD individuals, but not in four non-sensitized AD or three healthy donors (18). These experiments show altogether a clear-cut cross-reactivity between human and fungal allergen homologs and further a tight correlation between IgE and T cell responses, which appears to have clinical implications in allergic diseases (38, 39).

In a laborious approach T cell clones were generated from *Malassezia*-sensitized AD donors, from blood as well as from APT-lesions induced by Mala s13 or *Malassezia* extract. These clones did cross-react to both proteins and were assigned regarding the respective cytokine production to Th1, Th2, Th17, as well as Th22 T helper cell subsets (40). Later, our group described that hTrx upregulates the Th2 cytokine IL-13 in an IgE-dependent manner and showed further an impaired upregulation of IL-10 by hTrx in specifically sensitized AD patients (41). hTrx is known to act also as an alarmin, being secreted upon cellular stress (42). This suggests that the protein is often recognized by the immune system within danger situations, which may favor sensitization. Recent studies show that hTrx as well as Mala s13 are effectively bound by pattern recognition receptors on myeloid cells, and that hTrx directly induces pro-inflammatory responses that promote the survival of Th17 cells (Roesner et al., unpublished). Both crossreactivity as well as intrinsic properties of hTrx may therefore underlie the observed cellular and humoral responses.

Regarding the autoallergens that were identified by the human protein-directed approach by Natter et al. (21), α-NAC/Hom s2 as well as MICU1/Hom s4 have been shown to evoke IFN-γ responses from peripheral blood cells of sensitized donors. These reactions were found to be stronger compared to those by the classical pollen allergen Phl p1 (12, 43). MICU1/Hom s4, however, also promoted IFN-γ in healthy individuals, suggesting that the protein might activate parts of the innate immune system. The Th1-response by α-NAC/Hom s2 was later shown to be dependent on IL-12 and mediated through TLR-2 on monocytes, also describing an effect aside from the adaptive T cell response (44). Proliferation testing of T cells from the blood of sensitized donors, however, revealed that α-NAC/Hom s2 specifically triggered skin-homing T cells in AD patients. Generating subsequently α-NAC/Hom s2-specific T cell clones, CD4^+^ but interestingly also CD8^+^ T cells were could be established. While from lesional AD skin only 10% of T cell clones were CD8^+^, surprisingly 61% clones generated from the circulation of sensitized donors were found to be cytotoxic T cells (45). These did secrete IFN-γ and/or IL-4 as well as occasionally IL-17 upon autologous, specific re-stimulation.

The identification of autoreactive T cells homing to / infiltrating in the skin clearly indicates that these promote the pro-inflammatory milieu in the inflammatory response in affected patients. However, relatively low numbers of participants narrow the impact of these experiments.

Deeper insights come from approaches evoking skin lesions in patients' skin. Recombinantly produced human MnSOD has been applied to patient's skin in the context of an atopy patch test (APT) (8). This test aims for the late type (type IV-like) hypersensitivity skin reaction to protein allergens in a controlled fashion (46). This positive skin reaction can therefore be seen as a proof-of-concept-observation of the clinical relevance of a given sensitization. Within the EU, only those test substances are allowed nowadays to be applied on the skin, which have been produced under GMP conditions. This production process however is often financially not profitable which explains that the APT is generally not available as a routine diagnostic tool (47, 48).

Further deciphering of the T cell reactivity to autoallergens is achieved by identifying immunodominant epitopes within the amino acid sequence. T cells recognize linear epitopes that are presented by MHC complexes of antigen presenting cells. Breaking-down the amino acid sequence into immunodominant epitopes can therefore be achieved by applying single synthetic peptides in stimulation or binding assays. Therefore, candidate peptides are often produced in an overlapping fashion, covering the complete sequence of the protein of interest. A possibility to downsize these laborious approaches is to apply prediction algorithms upfront that identify MHC-binding motifs within the primary sequence [like SYFPEITHI (49) and consensus (50)]. For hTrx and α-NAC/Hom s2 candidate T cell epitopes were presented to PBMC of sensitized donors, which led to modest but measureable T cell proliferation in sensitized donors and finally to the identification of MHC-I and MHC-II epitopes (51, 52).

## Are Autoallergens Presented via MHC?—a Deeper Look Into the “Peptidome”

Cross-reactivity of T cells is nowadays an accepted immunological phenomenon. The specificity of the T cell is given by the highly diverse TCR, which is generated by the process of V(D)J recombination and can theoretically lead to 10^17^ different receptors (53, 54). However, the naïve T cell pool of a human being consists “only” of 10^7^ to 10^13^ different TCR (55–57), what raises the question how these are able to cover a virtually unlimited set of pathogenic molecules (>10^15^ possibilities from 20 proteogenic amino acids) (58). Recent studies showed that each TCR is able to get activated by millions of different peptide/MHC complexes (58). While at least two amino acids within an epitope were considered as nearly irreplaceable since serving as anchor positions for the MHC, also these have been shown to be variable to a certain extent (59). It is believed that the high variability is not leading to a complete autoimmune-chaos, since a) not every protein is present in every tissue, and b) not every possible combination is generated by the antigen-presenting cells. The question whether a putative immunodominant epitope is presented via MHC is therefore of central interest.

Meanwhile, harnessing the power of high-throughput proteomics, T cell epitopes presented by MHC-molecules on the surface of antigen-presenting cells can be identified by mass spectrometry. These immuno assays represent beautiful but laborious as well as money-consuming approaches enabling an objective view into what T cells might react to. However, caution has to be taken since donors differ regarding the MHC molecules and every MHC has different binding abilities, leading to a patient-specific set of presented epitopes for each antigen. This is usually taken into account by enlargement of the test cohort. Epitopes discovered to be presented in different donors are of special interest, since these may represent targets for vaccination or allergen immunotherapy. To date, most of these assays have been performed on cells derived from healthy donors to get a picture of the status quo of antigen presentation (60–63); but also data on specific diseases are available (64, 65). These studies are listed within the PRoteomics IDEntifications (PRIDE) database. The consortium Human Immuno-Peptidome Project (HUPO-HIPP) intends to define experimental standards, to connect labs generating these data, and to gather available information in order to display the sequences to scientists around the world on a single platform, the SysteMHCAtlas (66). This database harbors today data from 23 experimental approaches. Data mining in this archive reveals that autoallergens are indeed frequently presented. For example regarding the autoallergen α-NAC/Hom s2, 66 presented epitopes have been identified by mass spectroscopy. Interestingly, 49 of 66 are mapped within the sequence stretch α-NAC/Hom s2_181−209_ VKLVMSQANVSRAKAVRALKNNSNDIVNA, which has also been found to be immunogenic in our studies on autoallergic AD patients (51). Further, these data match our own findings, that MHC-I and MHC-II epitopes overlap in this region (52). Also epitopes of DSF70, Hom s3, and Hom s4 could be identified: Table 2 lists the most commonly identified epitopes and their appearances in the assays. Epitopes of hTrx were found in at least two studies (60, 67).

**Table 2:** Frequently found MHC-presented epitopes of exemplary autoallergens.

**Autoallergen**	**MHC class**	**Consensus sequence of overlapping epitopes**	**# of epitopes found**	**# of studies**	**HLA**
DSF70	I	ATASVNLKVSPK	62	8	A*03:01 A*68:01 A*11:01 A*31:01
		KVSQVIMEKSTMLY	61	8	A*01:01 A*03:01 A*68:01
		KAVDITTPKAA	12	4	A*03:01 A*11:01
		SVITQVLNK	10	4	A*03:01 A*68:01 A*11:01 A*30:01
α-NAC/Hom s2	I	ANVSRAKAVRAL	40	8	B*07:02 C*06:02 B*39:24
		QENTQTPTV	5	4	B*40:01 B*40:02 B*45:01
		KSKNILFVITK	4	1	A*11:01 B*57:01
	II	NVSRAKAVRALKNNSNDIVNA	9	1	n.a.
		EEVDETGVEVKD	3	1	n.a.
		LSQQAQLAAAEKF	2	1	n.a.
BCL7B/Hom s3	I	EEDSGAPPLKRF	9	6	B*44:02 B*44:03
		KVMAAIEKVRK	2	2	A*03:01
MICU1/Hom s4	I	RPTTGNTL	24	7	B*07:02
		VTASTGLLWK	7	5	A*03:01 A*11:01
		AELAVGSRW	12	6	B*44:02 B*44:03
	II	RSITPNEKQPEHLGLDQY	5	1	n.a.

*This list was extracted from the SysteMHCAtlas (66). This list is not exhaustive*.

These data support the probability of autoallergens to play a role in disease, since their epitopes are commonly presented. It appears even more compelling, that presented natural epitopes of α-NAC/Hom s2 have also been described to evoke pro-inflammatory responses in sensitized subjects.

## Autoreactive T Cell Receptors, a Matter of Specificity

MHC-multimers are further tools to investigate the question whether a certain epitope is recognized by T cells. These multimerized, labeled MHC/peptide-complexes have been shown to bind to matching T cells with strong specificity, allowing their enumeration and characterization (68). With MHC class I-multimers harboring α-NAC/Hom s2 epitopes, we observed specific staining of a subgroup of CD8^+^ T cells in patients that displayed detectable levels of specific IgE (51). This T cell fraction showed specific characteristics of effector/memory (T_EM_) of terminally differentiated effector T (T_EMRA_) cells, arguing for a contribution in an ongoing inflammatory process. Measuring cytokines secreted by these cells, we detected first of all IL-4, and further (but less) IFN-γ. While this phenotype, also termed T_c_2, is relatively uncommon in healthy donors, it reflects the cytokine milieu in AD. Again, quality and quantity of the immune response suggest a contribution to the disease pathogenesis.

Interestingly, the binding affinity of identified peptides to the MHC-I molecules, as well as the binding avidity of the tetramer to the TCR were both observed at a rather low level (51). Based on this, it could be hypothesized that autoallergen-specific T cells do not harbor perfectly matching T cell receptors (TCR). This might indeed be the case, since those would have been eradicated during negative selection in the thymus. The only possible explanation of autoreactivity in AD, where a fully functional thymus can be assumed, is that T cells with minimal autoreactive potential escape the negative selection. Upon encounter of the autoallergen within a highly inflammatory milieu, however, the suboptimal recognition may be sufficient to mount a pro-inflammatory response against self. The expression of a low avidity TCR can be seen as a major mechanism by which autoreactive T cells escape tolerance. Different studies have demonstrated that that autoreactive T cells that are not completely eliminated by negative selection due to low avidity are quiescent under steady-state conditions in the presence of their target, but during an infection they are able to respond to the respective antigen and differentiate into effector T cells and form memory T cells (69, 70). Interestingly, these low avidity T cells in contrast to high avidity T cells appear to persist without losing their self-destructive potential (70). Szomolay described that also other TCR that recognize self-antigens may do so with low avidity: The MART-1/melan-A antigen, which is specific for the melanocyte lineage and also found in normal skin, evokes T cell reactivity, but the respective TCR could be shown to react much better to other (hypothetical) peptides (71). Contrary to that, TCR that recognize pathogen epitopes (which are not displayed during negative selection) are often perfectly suitable to the respective epitope (71). These findings match to the idea of Wooldridge et al., who describe that TCR do not recognize one single peptide epitope, but broader signatures and many different epitopes (58, 59).

## Conclusion

Historical observations in the patients' skin argue for a pathogenic role of autoallergy in AD. A high number of IgE-reactive auto-antigens have been identified by now, what appears to be specific for AD. These bear the potential to mount an inflammatory response. For several autoantigens immune-stimulatory functions have been described, and different receptors have been shown to bind self-antigens. The limited success of anti-IgE treatment may underlie the fact that AD is strongly driven by specific T cell responses. However, a subgroup of patients may indeed benefit from this therapeutic (4, 72, 73). T cells recognizing autoallergens have been shown to be of effector subtype, to home to and infiltrate the skin. These have been observed to respond with different cytokines involving IFN-γ, IL-4, and IL-13, but also IL-17 and IL-22. Further *in vitro* data were generated on T cell epitopes. Although this is known to bear certain risks, recent data corroborate these findings. Difficulties in detecting the immune responses may most probably result from the fact that autoallergens do not harbor perfectly matching T cell epitopes, since such T cells are eliminated efficiently in the thymus during maturation. Nevertheless, reviewing data that are available today, there is no plausible reason to deny an impact of autoreactivity in AD.

## Author Contributions

All authors listed have made a substantial, direct and intellectual contribution to the work, and approved it for publication.

### Conflict of Interest Statement

The authors declare that the research was conducted in the absence of any commercial or financial relationships that could be construed as a potential conflict of interest.
